# Incubation Temperature Affects Duckling Body Size and Food Consumption Despite No Effect on Associated Feeding Behaviors

**DOI:** 10.1093/iob/obaa003

**Published:** 2020-02-05

**Authors:** S F Hope, R A Kennamer, A T Grimaudo, J J Hallagan, W A Hopkins

**Affiliations:** 1 Department of Fish and Wildlife Conservation, Virginia Tech, Blacksburg, VA, USA; 2 Savannah River Ecology Laboratory, University of Georgia, Aiken, SC, USA

## Abstract

Developmental conditions can have consequences for offspring fitness. For example, small changes (<1°C) in average avian incubation temperature have large effects on important post-hatch offspring phenotypes, including growth rate, thermoregulation, and behavior. Furthermore, average incubation temperatures differ among eggs within the same nest, to the extent (i.e., >1°C) that differences in offspring phenotypes within broods should result. A potential consequence of within-nest incubation temperature variation is inequality in behaviors that could cause differences in resource acquisition within broods. To investigate this, we incubated wood duck (*Aix sponsa*) eggs at one of two ecologically-relevant incubation temperatures (35°C or 36°C), formed mixed-incubation temperature broods after ducklings hatched, and conducted trials to measure duckling behaviors associated with acquisition of heat (one trial) or food (three trials). Contrary to our predictions, we found no effect of incubation temperature on duckling behaviors (e.g., time spent occupying heat source, frequency of feeding bouts). However, we found evidence that ducklings incubated at the higher temperature consumed more food during the 1-h feeding trials, and grew faster in body mass and structural size (culmen and tarsus) throughout the study, than those incubated at the lower temperature. Apparent food consumption during the trials was positively related to culmen length, suggesting that differences in food consumption may be driven by structural size. This could result in positive feedback, which would amplify size differences between offspring incubated at different temperatures. Thus, our study identifies incubation temperature as a mechanism by which fitness-related phenotypic differences can be generated and even amplified within avian broods.

## Spanish Translation

**Translated by Jazel Ouled-Cheikh Bonan:**

**Título**

La temperatura de incubación afecta el tamaño corporal y el consumo de alimentos de los patos juveniles a pesar de no tener ningún efecto sobre los comportamientos alimentarios asociados

**Resumen**

Las condiciones del desarrollo pueden tener consecuencias para la eficacia biológica de la descendencia. Por ejemplo, en aves, pequeños cambios (<1°C) en la temperatura promedio de incubación tienen grandes efectos sobre los fenotipos importantes de la descendencia después de la eclosión, incluida la tasa de crecimiento, la termorregulación y el comportamiento. Además, las temperaturas promedio de incubación difieren entre los huevos dentro del mismo nido, de forma que deberían dar lugar a diferencias en los fenotipos de descendencia dentro de la nidada. Una posible consecuencia de la variación de la temperatura de incubación dentro del nido es la diferencia en los comportamientos que podrían causar contrastes en la adquisición de recursos entre polluelos. Para investigar esto, incubamos huevos de pato joyuyo (*Aix sponsa*) a diferentes temperaturas de incubación ecológicamente relevantes (35 o 36°C). A continuación, después de la eclosión de los polluelos, formamos grupos mixtos con respecto a la temperatura de incubación y realizamos ensayos para medir los comportamientos de los polluelos asociados con la adquisición de calor (un ensayo) o comida (tres ensayos). Contrariamente a nuestras predicciones, no encontramos ningún efecto de la temperatura de incubación en los comportamientos de los polluelos (p. Ej., tiempo dedicado a ocupar la fuente de calor, frecuencia de las peleas relacionadas con la comida). Sin embargo, encontramos evidencia de que los polluelos incubados a la temperatura más alta consumieron más alimentos durante las pruebas de alimentación de 1 hora, y crecieron más rápido en masa corporal y tamaño estructural (culmen y tarso) durante todo el estudio, que los incubados a la temperatura más baja. El consumo aparente de alimento durante los ensayos se relacionó positivamente con la longitud del culmen, lo que sugiere que las diferencias en el consumo de alimentos pueden deberse al tamaño estructural. Esto podría dar como resultado una retroalimentación positiva, lo que amplificaría las diferencias de tamaño entre las crías incubadas a diferentes temperaturas. Por lo tanto, nuestro estudio identifica la temperatura de incubación como un mecanismo por el cual se pueden generar diferencias fenotípicas relacionadas con la eficacia biológica dentro de las nidadas e incluso amplificarlas.

**Palabras clave**

*Aix sponsa*, entorno de desarrollo temprano, consumo de alimentos, crecimiento, efecto parental, interacciones fraternales.

## French Translation

**Translated by Mathias Dezetter and Frédéric Angelier:**

**Titre**

La température d’incubation affecte la taille corporelle et la consommation de nourriture des canetons malgré l’absence d’effet sur les comportements alimentaires associés

**Résumé**

Les conditions de développement peuvent avoir des conséquences sur la valeur sélective de la progéniture. Par exemple, chez les oiseaux, de faibles changements de la température d’incubation moyenne (<1°C) ont des effets forts sur des paramètres phénotypiques importants de la progéniture après l’éclosion, notamment sur le taux de croissance, la thermorégulation et le comportement. De plus, les températures d’incubation moyennes diffèrent généralement entre les œufs d’une même nichée, ce qui devrait également engendrer des différences phénotypiques au sein de la couvée. De telles variations de la température d’incubation au sein des nids pourraient conduire à des différences comportementales, notamment en ce qui concerne l’acquisition des ressources des jeunes éclos. Nous avons incubé des œufs de canard carolin (*Aix sponsa*) à deux températures contrastées, pertinentes écologiquement (35 ou 36°C). Après l’éclosion, les canetons incubés aux deux températures ont été regroupés dans des lots mixtes et des tests comportementaux ont été menés: un test d’accès à la chaleur, et trois tests d’accès à la nourriture. Contrairement à nos prédictions, nous n’avons trouvé aucun effet de la température d’incubation sur les comportements des canetons (i.e., le temps passé à occuper la source de chaleur, la fréquence d’alimentation). Cependant, les canetons incubés à une température plus élevée consomment plus de nourriture pendant les tests d’alimentation d’une heure, et grandissent plus rapidement (masse corporelle et taille structurelle (culmen et tarse)) que ceux incubés à température plus basse. La consommation alimentaire mesurée au cours des tests était positivement liée à la longueur du culmen, ce qui suggère que les différences de consommation alimentaire peuvent être à l’origine des différences de taille observées. Cela pourrait entraîner un effet sur la croissance, qui amplifierait les différences de taille entre les progénitures incubées à des températures contrastées. Notre étude démontre que la température d’incubation peut générer des différences phénotypiques au sein des couvées d’oiseaux, et même les amplifier.

**Mots-clés**

*Aix sponsa*, environnement de développement précoce, consommation alimentaire, croissance, effet parental, interactions fraternelles

## Portuguese Translation

A temperatura de incubação afeta o tamanho corpóreo e o consumo de alimento em filhotes de pato-carolino, apesar de nenhum efeito sobre os comportamentos alimentares associados

**Resumo**

As condições de desenvolvimento durante a incubação podem ter consequências para a aptidão da prole. Por exemplo, pequenas alterações na temperatura média (< 1°C) de incubação das aves têm grandes efeitos sobre fenótipos importantes da prole pós-eclosão, incluindo taxa de crescimento, termorregulação e comportamento. Além disso, as temperaturas médias de incubação diferem entre os ovos no mesmo ninho, podendo igualmente resultar em alterações fenotípicas sobre a prole. Tal variação de temperatura dentro do ninho durante o período de incubação acarreta diferenças comportamentais entre os filhotes, sobretudo quanto à obtenção de recursos pela ninhada. Para investigar tais efeitos, ovos de patos-carolinos (*Aix sponsa*) foram incubados em duas temperaturas ecologicamente relevantes (35 ou 36°C). Após a eclosão, os filhotes incubados em ambas temperaturas foram reagrupados em lotes mistos, e medidas de comportamento foram realizadas: um teste quanto à aquisição de calor e três testes associados à obtenção de alimento. Ao contrário de nossas predições, não encontramos efeito da temperatura de incubação no comportamento dos patinhos (e.g., tempo gasto ocupando fonte de calor, frequência de registro de alimentação). No entanto, os filhotes de patos incubados em temperaturas mais altas ingeriram mais alimento durante os testes de alimentação de uma hora, e cresceram mais rapidamente em massa corporal e tamanho estrutural (cúlmen e tarso) do que aqueles incubados em temperaturas mais baixas. Ademais, o consumo alimentar foi positivamente relacionado ao comprimento do cúlmen, sugerindo que as diferenças na ingestão de alimento podem ser causadas pelo tamanho estrutural. Isso resultaria em um *feedback* positivo, o que amplificaria as diferenças de tamanho entre os filhotes incubados em diferentes temperaturas. Desta forma, nosso estudo identifica a temperatura de incubação em aves como mecanismo capaz de gerar, e até mesmo ampliar, diferenças fenotípicas das ninhadas.

**Palavras-chave**

*Aix sponsa*, ambiente inicial de desenvolvimento, consumo de alimentos, crescimento, efeito parental, interações entre irmãos

## Introduction

Across taxa, parents can have non-genetic effects on the phenotype and fitness of their offspring. Parental effects such as nest site choice, differential allocation of hormones/nutrients to embryos, food provisioning, and grooming have long-lasting consequences for offspring phenotype (Bernardo 1996; Lindström 1999; Mousseau and Fox 1998). In addition to affecting offspring morphology, physiology, and behavior, parents can also affect offspring by influencing the potential for differential resource acquisition within the brood/litter. For example, if parents distribute resources (e.g., nutrients and hormones) unequally among embryos within the same brood/litter, it can create variation in individual offspring phenotypes, with consequences for offspring ability to acquire additional resources (Eising and Groothuis 2003; Müller et al. 2012; Correa et al. 2013). Those individuals that are able to maximize their resource acquisition, either from their parent or from the environment, will have an advantage. In turn, this can either amplify or reduce differences in phenotype and survival among offspring within a brood (Drummond et al. 2000; Groothuis et al. 2005; Muller and Groothuis 2013; Hofer et al. 2016). Understanding how parents can influence offspring fitness by creating differences among siblings is necessary for a comprehensive understanding of the consequences of parental effects.

In oviparous species, one of the most important ways that parents can affect offspring phenotype is through egg incubation temperature (Deeming and Ferguson 1991; Hepp et al. 2015). In most oviparous reptiles, amphibians, fish, and invertebrates, egg temperatures are largely determined by parental nest site choice and the external environment (e.g., Shine and Harlow 1996; Kolbe and Janzen 2002; Thompson et al. 2018b), which can affect offspring developmental rate, hatch success, morphology, growth rate, metabolism, locomotor performance, sex ratio, and behavior (e.g., Deeming and Ferguson 1991; Sakata and Crews 2003; Watkins and Vraspir 2006; Booth 2006; Putz and Crews 2006; Amiel and Shine 2012; Réalis-Doyelle et al. 2016; Uriarte et al. 2016; Siviter et al. 2017; Ross-Gillespie et al. 2018; While et al. 2018; Mueller et al. 2019). In contrast, most birds actively heat eggs through contact-incubation, which is an energetically costly and time-consuming aspect of parental care (Tinbergen and Williams 2002; Nord and Williams 2015). Thus, avian parents must tradeoff time and energy between incubation and self-maintenance. Furthermore, incubation behavior varies depending on factors such as weather, parental body condition, and clutch size, and this leads to differences in egg incubation temperatures among nests in the same population, and even among different breeding attempts of the same individual (Aldrich and Raveling 1983; Haftorn and Reinertsen 1985; Conway and Martin 2000; Ardia et al. 2010; Nord et al. 2010; Coe et al. 2015; Hope et al. 2018a). This temperature variation is important for the offspring because, like non-avian taxa, small differences in average egg incubation temperature (<1°C) have large effects on post-hatch avian offspring phenotypic traits (DuRant et al. 2013b), such as growth rate (DuRant et al. 2010; Nord and Nilsson 2011; Wada et al. 2015; Ospina et al. 2018), thermoregulatory ability (DuRant et al. 2013a), hormone levels (DuRant et al. 2010, 2014; Wada et al. 2015), and proactive/reactive behavior (Bertin et al. 2018; Hope et al. 2018b). Furthermore, incubation temperature is related to survival (Hepp and Kennamer 2012; Berntsen and Bech 2016; Nord and Nilsson 2016), suggesting that these phenotypic differences have fitness consequences.

Incubation temperature is an aspect of the early developmental environment that also has the potential to influence within-brood variation in offspring phenotypes. In oviparous taxa where parents do not engage in contact-incubation, egg temperatures can differ substantially within nests. For example, egg temperatures can be vertically stratified within turtle nest chambers, leading to different phenotypes produced at the warmer and more variable top of the nest compared to the bottom of the nest (Thompson et al. 2018a, 2018b). In contrast, most avian parents that actively incubate their eggs attempt to mitigate thermal variance within the clutch by rotating and repositioning their eggs throughout incubation (Stewart 1971; Boulton and Cassey 2012). However, recent evidence shows that despite these efforts by avian parents, average incubation temperatures can substantially differ among eggs within avian nests (Reid et al. 2000; Beatty 2015; Hope et al. 2018a). For example, in wood ducks (*Aix sponsa*), average egg temperatures throughout the entire incubation period differ sufficiently to create broods containing individuals with different phenotypes (i.e., >1°C; Hope et al. 2018a).

Within-nest variation in average incubation temperature could result in differential ability of siblings to acquire resources because incubation temperature can produce differences in traits that influence competitive ability such as offspring size, hormone levels, and proactive/reactive behavior (Greig‐Smith 1985; Oddie 2000; Kitaysky et al. 2001; Ward et al. 2004; Cole and Quinn 2012; Ruppli et al. 2012). Until now, studies investigating the influence of parental effects on differential resource acquisition within avian broods have focused on how hormone allocation to embryos and hatching asynchrony can influence offspring competition for resources from the parent, and subsequently lead to differential growth and survival within broods (Schwabl 1996; Ostreiher 1997; Krebs et al. 1999; Ploger and Medeiros 2004; Morandini and Ferrer 2015). No studies have heretofore examined the influence of within clutch variance in thermal conditions on relative competitive abilities of siblings. If incubation temperature affects the ability of avian offspring to access resources, this would reveal a previously unrecognized way by which differences in resource acquisition among siblings could be created within avian broods.

To investigate if differential resource acquisition within avian broods could be a consequence of variation in average incubation temperature among eggs within nests, we conducted an experiment to determine whether differences in incubation temperature affect the ability of wood duck ducklings to gain access to heat and food sources. We selected wood ducks as a model because they are among the most well studied wild birds in regard to the effects of incubation temperature on offspring phenotype (DuRant et al. 2013b), and experience variable average egg temperatures both among and within nests in the field (Hope et al. 2018a). We incubated wood duck eggs at two different average temperatures and formed mixed-incubation temperature broods. Then, we conducted one trial to measure behaviors associated with the ability to gain access to a source of heat and three trials to measure behaviors associated with the ability to gain access to food. We conducted three feeding trials because we were interested in how resource acquisition could be influenced by different environmental contexts (e.g., familiar vs. unfamiliar environment). We measured duckling body mass before and after feeding trials to estimate food consumption and verify that feeding behavior correlated with food acquisition. We also measured duckling body mass, tarsus length, and culmen length throughout the experiment to determine whether differences in resource acquisition could either amplify or reduce morphological differences within broods.

We had two alternative predictions. First, because higher incubation temperatures produce ducklings with faster growth rates (DuRant et al. 2010), greater locomotor abilities (Hopkins et al. 2011), and possibly a greater probability of survival (Hepp and Kennamer 2012), we predicted that ducklings incubated at higher temperatures would be physically advantaged (e.g., larger, faster, and stronger) and thus would outperform ducklings incubated at the lower temperature, regardless of the environmental context. If this is the case, we would expect that differences in incubation temperature within broods would amplify phenotypic (morphological) differences among offspring. Alternatively, because lower incubation temperatures produce ducklings with slower growth rates (DuRant et al. 2010), higher metabolic rates during a thermal challenge (DuRant et al. 2012b) and weaker thermoregulatory abilities (i.e., greater reduction in body temperature during a thermal challenge; DuRant et al. 2013a), it is possible these ducklings may be more inclined to take risks (e.g., forage in a risky environment) or make a greater effort (e.g., push their way to a warmer position in the brood) to meet their nutritional and thermoregulatory needs. Indeed, ducklings incubated at a lower temperature display more proactive (i.e., risky, bold, and exploratory) behaviors than those incubated at higher temperatures (Hope et al. 2018b). Because there is evidence that proactive behavior is positively related to competitive ability (Ward et al. 2004; Cole and Quinn 2012), we predicted that it may be possible for ducklings incubated at the lower temperature to acquire equivalent or more resources than those incubated at higher temperatures, especially within a novel environmental context. If this is the case, it could reveal a way by which ducklings incubated at lower temperatures could achieve morphology and physiology (e.g., body size and maintenance of body temperature) similar to those incubated at warmer temperatures, through changes in their behavior.

## Methods

### Study species

The wood duck (*A.* *sponsa*) is a dabbling duck that is widely distributed throughout North America and nests in tree cavities and nest boxes (Hepp and Bellrose 2013). Wood ducks lay an average of 12 eggs per clutch (Bellrose and Holm 1994). However, conspecific brood parasitism is common both in natural cavities (Roy Nielsen et al. 2006) and nest boxes (Semel and Sherman 1986; Semel et al. 1988), and thus clutches can reach >40 eggs in some populations (Morse and Wight 1969; Eadie et al. 1998). Only the females incubate (Hepp and Bellrose 2013). The incubation period lasts ∼30 days and females usually take two 1–2 h recesses per day to forage (Manlove and Hepp 2000). Incubation temperature affects a wide array of traits in wood ducks (DuRant et al. 2013b). Average incubation temperature varies both among and within nests in the field, and average egg temperatures can differ by >3°C among different eggs within the same clutch (Hope et al. 2018a). Thus, natural wood duck broods consist of ducklings that have hatched from eggs that were incubated at different average temperatures.

Wood duck ducklings are precocial and leave the nest within 24 h of hatching. Ducklings stay with their mother for ∼5 weeks, and the mother provides warmth, guides ducklings to sources of food, and provides protection from predators (Bellrose and Holm 1994). However, ducklings are not completely dependent on their mother. They can feed themselves once they leave the nest, and can seek other ducklings in the same brood to huddle with for warmth. Indeed, duckling broods that are separated from their mother are known to sometimes survive in the wild (Bellrose and Holm 1994). Ducklings spend much of their time in the water, but also frequently spend time on land to warm themselves, especially in early spring when the water is cold. Furthermore, because hens do not always nest directly over a body of water, it is common for ducklings to travel long distances on land to reach a body of water (Bellrose and Holm 1994). Ducklings are most vulnerable to starvation, cold temperatures, and predators during the first 2 weeks of life (Bellrose and Holm 1994). A large proportion (50–75%) of ducklings die before they can fly (∼Day 60), and 90–99% of these mortalities occur within the first 1–2 weeks of life (McGilvrey 1969; Sedinger et al. 2018). Thus, duckling behaviors related to seeking warmth or food during this early-life period are critical for survival.

### Egg collection and incubation

We collected eggs from a wood duck population breeding in nest boxes, which have been maintained for >35 years, on the Department of Energy’s Savannah River Site (SRS) in South Carolina (33.1°N, 81.3°W) from February 29 to March 16, 2016. We checked nest boxes daily on 12 ephemeral wetlands, marked each egg for lay –date and –order, and collected up to 10 eggs from each nest before the hen began to incubate. We collected 200 eggs from 32 nests, with an average of 6 eggs per nest (range 1–10 eggs), for use in this experiment and a concurrent experiment (Hope et al. 2019). We replaced eggs with wooden eggs to prevent hen abandonment (Hepp et al. 1987), and transported the unincubated eggs to Blacksburg, VA, at room temperature. We held eggs at room temperature, rotating them twice daily, for ≤10 days before beginning incubation (mean ± standard deviation [SD] holding time = 6.9 ± 1.8 days; range = 4–10 days). Avian embryos do not begin developing when held at room temperature (i.e., below physiological zero; Webb 1987), and keeping wood duck eggs in this way before beginning incubation does not affect hatchability (Walls et al. 2011).

We then incubated eggs for the entire incubation period in Grumbach incubators (model BSS 420, Asslar, Germany) at two different overall mean temperatures: 35.0°C and 36.0°C, within the natural range for wood ducks (Hepp et al. 2006; Hope et al. 2018a). We chose these two temperatures because they have been shown to produce a wide array of differences in duckling traits in previous studies (DuRant et al. 2013b), such as different growth rates (DuRant et al. 2010), metabolic rates during a thermal challenge (DuRant et al. 2012b), and ability to maintain body temperature during a thermal challenge (DuRant et al. 2013a). Further, a difference of 1°C in average incubation temperature among eggs within the same clutch is realistic and likely common in the wild (Hope et al. 2018a). We distributed eggs from the same nest and the same lay date evenly between treatments. We programmed incubators to have two 75 min cool-down periods at 0815 and 1830 h to simulate hens leaving the nest for foraging (Manlove and Hepp 2000), but incubators maintained the above-mentioned overall mean temperatures, as measured by two iButtons^®^ (Hygrochron, Maxim Integrated) in each incubator. We kept the average humidity for both incubators between 60% and 65%. In total, we used 120 ducklings from 32 clutches in this study. We tested all ducklings in every trial. Because some ducklings died before the end of the experiment (*n *=* *12), we state specific sample sizes for each trial in “Results” section.

### General husbandry

Upon hatching, we recorded date/time, and weighed and color-banded ducklings. We checked the hatcher every 2 h or video-recorded it during longer time periods to ensure that our hatch times (and thus, duckling ages) were accurate. As part of a different study, ducklings used in the current trials first performed a test of their ability to exit the nest within 24 h of hatching, using a nest box set-up in the laboratory and playing a wood duck hen call as a stimulus for 30 min (as described in Hope et al. 2019). Then, we housed ducklings in pairs or groups of three (mixed-incubation temperatures) in plastic cages assembled in a rack system. Each cage had a 50 W infrared light and ad-lib food (DuMOR Chick Starter/Grower 20% Feed, Tractor Supply Co.^®^) and water. To allow for individual identification during all behavioral trials, we marked ducklings with numbers on their heads and dots on their backs using non-toxic white correcting fluid (Supplementary Fig. S1A).

Once ducklings were 4 days old, we formed broods of six ducklings and housed them in semi-outdoor aviary rooms. Because seven ducklings died between the heat trial (Days 2–3; see “Heat trial” section) and brood formation in aviaries, we rearranged the brood composition at this point in the study. However, once in aviaries, individuals never changed broods. Broods consisted of three ducklings from each incubation temperature. We chose a brood size of 6 because it is a realistic size for wood ducks in the wild, and it was small enough to both maximize sample size and be logistically feasible given the difficulty of attaining sufficient hatching synchrony using artificial incubation. In 14 out of 57 feeding trials, we used a brood of five because some ducklings (*n* = 5) died after brood formation in aviaries (see “Feeding trials in three contexts” section for sample sizes). The aviary rooms (5.5 m × 2.5 m) were semi-outdoor, with mesh on three walls, covering the top half of each wall. Each room had a 100 W infrared heat lamp, food, and water. We assembled the feeding area specifically to acclimate ducklings to eating from a dish similar to those used in the familiar environment and novel object feeding trials (see below). The dish was a plastic cylinder with multiple openings, so food was replenished as the ducklings fed (Supplementary Fig. S1B). There was also a metal grate (40 cm × 42 cm) underneath the food, so that spilled food was not accessible (Supplementary Fig. S1C).

We measured duckling body mass and culmen length on Days 0, 2, 4, 6, 8, and 10, and tarsus length on Days 0, 3, 6, 8, and 10. Culmen length is the distance from the tip of the bill to the edge of the skull, and we took this measurement because we predicted that it could influence food consumption. We measured tarsus in triplicate and took the average of these measurements. Tarsus length is a common structural measure in birds and is the distance between the intertarsal joint of the leg and the juncture between the tarsometatarsus and the third digit of the foot. After all trials were complete, we euthanized ducklings using carbon dioxide asphyxiation followed by cervical dislocation, and determined sex by inspecting both external genitalia and internal gonads. All procedures were approved by the Institutional Animal Care and Use Committee (#15-009).

### Heat trial

When ducklings were 2–3 days old, we conducted a trial to assess ability to gain access to a concentrated heat source. For each trial, we transported six ducklings (three high temperature and three low temperature) from their cages, one brood at a time, to the trial arena (Supplementary Fig. S2). We conducted trials on 19 broods with six ducklings each. We conducted trials in the morning, starting between 0508 and 0617 h, and in an air-conditioned room (mean ± SD room temperature = 14.9°C ± 1.4°C; range = 12.3°C–17.4°C) so that the cold room temperature would encourage ducklings to seek the heat source and induce huddling. The precise boundaries of the thermoneutral zone of wood duck ducklings are not known (DuRant et al. 2012b), but the room temperature was below the lower critical temperature of other young dabbling ducks (e.g., mallard and Eurasian teal; 32°C; Koskimies and Lahti 1964). We recorded the temperature of the room before each trial. The arena was a circular (diameter = 50 cm) wooden platform with 50 cm walls and two cameras (GoPro^©^) mounted above to record behaviors. We suspended one 50 W infrared heat lamp above the arena and fitted it with metal flashing so only a small, concentrated amount of heat was emitted into the center of the arena. We fitted another 50 W infrared heat lamp beneath the arena to emit heat in the exact spot as the suspended heat lamp. We laid a piece of mesh over the heated spot (diameter = 4 cm). This spot of direct heat was about 35°C, and there was a considerable drop in heat in the spaces farther away from the heat source (e.g., the temperatures at 4, 8, and 15 cm away from the center of the heat source were ∼21°C, 19°C, and 17°C, respectively; see Supplementary Fig. S3). Thus, we predicted that ducklings would seek this heat source, which was not large enough to heat all ducklings. We allowed ducklings to acclimate in the arena for 15 min without the heat lamps on. After 15 min, we turned the heat lamps on remotely, and the trial lasted for 45 min.

We drew concentric circles on the floor of the arena to quantify duckling behavior. We analyzed videos and recorded the position of each duckling for each minute of the trial, starting when the light turned on, using a scan sampling approach, which is where the behavior of all members in a group are recorded at predetermined time-intervals (Altmann 1974). Position 1 indicated that the duckling was in the heat spot, the next concentric circle was recorded as Position 2, and so on, until Position 12. We determined position based on in which circle the majority of the duckling’s body was located. We also recorded the latency of each duckling to step onto the heat spot. Several broods did not huddle under the heat source (4 out of 19 broods), and instead, huddled in a different part of the arena. Thus, we also quantified the number of ducklings that were directly surrounding (i.e., making direct contact) each duckling for each minute of the trial, where a higher number indicated a warmer location. We then calculated the average position, average number of surrounding ducklings, number of minutes spent directly under the heat source (i.e., Position 1), and number of minutes spent within a 6 cm radius from the center point of the heat source (i.e., either Position 1, 2, or 3) for each duckling for the entire trial.

### Feeding trials in three contexts

Because ducklings incubated at different temperatures exhibit different exploratory and boldness behaviors (Hope et al. 2018b), and these behaviors are related to competitive ability in other species (Ward et al. 2004; Cole and Quinn 2012), we conducted feeding trials in three different contexts (novel environment, familiar environment, and novel object; see sections below). These trials were conducted after broods had already been formed and housed in aviary rooms, and ducklings were 6–11 days old. We conducted each trial on 19 broods consisting of either five or six ducklings, with two to three high temperature-incubated ducklings and two to three low temperature-incubated ducklings (number of broods consisting of five ducklings: novel environment feeding trial = 3; familiar environment feeding trial = 5; novel object feeding trial = 6). We conducted all trials in the same order for each brood to keep any effect of one trial on the behavior during another trial consistent among broods, similar to many other behavioral studies (e.g., van Oers et al. 2004; Bertin and Richard-Yris 2005; Reyes-Meza et al. 2011; Butler et al. 2012; Pittet et al. 2012, 2014). To stimulate feeding during the trials, we removed food from the aviary room (but water remained) 10 h 30 min before each trial would start the next morning. We recorded the temperature of the trial room before the start of each trial (for all trials: mean ± SD = 14.3°C ± 2.5°C; range = 7.9°C–17.4°C). Trials lasted 1 h and there was enough food in each trial so that ducklings could eat during the entire hour. We defined a feeding bout as a discrete up-and-down head movement into and out of the food dish.

To verify that feeding behavior was related to food consumption, we measured duckling body mass immediately before (fasted) and immediately after each of the three trials in order to calculate the change in body mass. Because we used the change in duckling body mass as a proxy of food consumption, we cannot discount the possibility that this measurement is confounded by digestive ability of ducklings or defecation rate. However, it is unlikely that ducklings would have fully digested the food ingested during the 1 h trial, so body mass differences should not reflect the ability to convert food to body mass or excrement. In support of this, feeding behavior (*z*-score; see “Statistical analyses” section) was positively related to apparent food consumption in all three feeding trials (all *P *≤* *0.012, all *r* ≥ 0.29), which verifies that ducklings that were quickest to feed and fed most frequently, as determined by the behaviors we measured, also consumed the most food.

### Novel environment feeding trial

The purpose of this trial was to investigate whether incubation temperature affected the ability of ducklings to acquire food while in a novel environment. Testing individual behavior in a novel environment could reveal the likelihood that the individual would explore new areas and take advantage of new foraging opportunities in unknown or risky environments in the wild (Koolhaas et al. 1999; Sih et al. 2004). When ducklings were 6–7 days old, we transported broods from their aviary rooms to the novel environment feeding trial arena (Supplementary Fig. S4). This arena was in a separate aviary room that was set-up in a different way than the home aviary rooms and thus was a novel environment. The arena (2.0 m × 2.5 m) had gridlines taped to the floor (forming squares with the dimensions: 0.25m× 0.25m), and 18 small food dishes spaced evenly throughout and secured to the ground. The dishes were small so that only one duckling could eat at a time. We placed a potted plant in front of each food dish so that the dishes were not immediately visible to ducklings. We started trials between 0555 and 0831 h. We placed broods under a bucket to acclimate in the dark for 5 min. Then, we remotely lifted the bucket, which allowed ducklings to explore and forage for 1 h. We mounted cameras (GoPro^©^) above the arena to record duckling behavior. From the videos, we quantified the latency to first feed, the number of unique dishes visited (i.e., if the duckling visited the same dish twice, the dish was not counted twice), the number of non-unique dishes visited (i.e., if the duckling visited the same dish twice, the dish was counted twice), the total number of feeding bouts (possible to have multiple feeding bouts per dish), and the total time spent at the dishes for each duckling.

### Familiar environment feeding trial

The purpose of this trial was to investigate whether incubation temperature affected the ability of ducklings to acquire food in a familiar environment. This trial can reveal how individuals might forage in a known, perceived safe, environment in the wild. We conducted the familiar environment trial when ducklings were 8–9 days old, and conducted it in the home aviary room (i.e., familiar environment) that the duckling broods had lived in since Day 4. We began trials between 0655 and 0722 h. On the morning of the trial, we replaced the metal grate below the food dish with one that had gridlines (forming squares with the dimensions: 10.0 cm× 10.5 cm) drawn on it and gave ducklings a food dish that was similar to the dish that they were accustomed to but had only one opening instead of four (Supplementary Fig. S5A), and thus only one duckling could eat at a time. The opening on the food dish was covered by a piece of plastic attached to a string. After the trial was set-up, we gave ducklings 15 min to re-acclimate and then we pulled the string from outside of the aviary room to reveal the food. We gave ducklings 1 h to access the food. We video-recorded trials and determined the latency to first feed, the latency to first enter the feeding area (metal grate), the total number of feeding bouts, the total number of times a duckling entered the feeding area, and the total amount of time spent in the feeding area for each duckling.

### Novel object feeding trial

The purpose of this trial was to investigate whether incubation temperature affected the ability of ducklings to acquire food that was in a familiar environment, but had a novel object near it. An individual that continues to forage in the presence of a novel object may be more likely to find and take advantage of novel food sources in the wild, or “innovate” to acquire a food source in a novel way (Kurvers et al. 2010; Overington et al. 2011). We conducted the novel object feeding trial when ducklings were 10–11 days old in the home aviary room (i.e., familiar environment), in which the duckling broods had lived since Day 4. We began trials between 0648 and 0716 h. This trial was the same as the familiar environment trial, with two exceptions. First, there was a novel object (9 cm tall pink plastic cone; Supplementary Fig. S5B) placed in front of the food dish. Second, a cardboard box (30 cm × 30 cm × 30 cm) covered both the food dish and the novel object during the acclimation period (15 min), and we lifted this box remotely by a string when the trial began. Through video analysis, we quantified the same behaviors as in the familiar environment trial for each duckling.

### Statistical analyses

Because we measured four to five behaviors for each trial, we condensed behavioral measures using *z*-score analysis (Guilloux et al. 2011; Labots et al. 2018; Hope et al. 2018b). For each individual behavior recorded, we calculated a *z*-score by subtracting the value from the mean value of all individuals for that behavior, divided by the SD. We calculated each *z*-score so that a higher value indicated a more active behavior (e.g., quicker to go to the heat source, quicker to begin feeding, more feeding bouts). We then calculated the average *z*-score of each individual for each trial, resulting in one *z*-score per individual per behavioral trial. The *z*-score for the heat trial included the average position, number of minutes spent directly under the heat (i.e., Position 1), number of minutes spent within 6 cm of the center point of the heat source (i.e., either Position 1, 2, or 3), and the latency to first go under the heat. A larger *z*-score indicated that a duckling was quicker to go to and spent more time under the heat source. Ducklings that did not spend any time under the heat source, but were within broods where at least one duckling spent time under the heat source, were included and given a latency of 45 min (i.e., the length of the trial; *n* = 3 ducklings). Broods in which no ducklings went under the heat source were excluded (*n* = 4 broods) from this analysis. The *z*-score for the novel environment feeding trial included the number of unique dishes visited, number of non-unique dishes visited, latency to feed, total number of feeding bouts, and the total time spent at the dishes. The *z*-scores for both the familiar environment and novel object feeding trials included the latency to feed, latency to enter the feeding area, total number of times in the feeding area, total number of feeding bouts, and total amount of time spent in the feeding area. For all feeding trials, a larger *z*-score indicated that the duckling was quicker to begin feeding and fed more frequently.

We used R version 3.5.1 (R Core Team 2018) for all analyses. We use the *lme4* package (Bates et al. 2015) for all linear mixed effects models (*lmer*) and report *P*-values using Type III Wald chi-square tests using the *Anova* function of the *car* package (Fox and Weisberg 2011). We reduced all models by using stepwise backward elimination of non-significant terms. Because we used the same ducklings in four different behavioral trials, we used a Bonferroni correction (α = 0.05/4 = 0.0125) and thus set significance at *P *<* *0.0125 for all models investigating behavioral endpoints. Here, we only report terms that were retained in the models, but we report all full and final models in Supplementary Information. We examined histograms of residuals, predicted versus residuals plots, and normal quantile plots to ensure that all models met the assumptions of normality and homoscedasticity, and used the *vif* function of the *car* package to ensure that models did not have multicollinearity among predictors.

To examine the ability to gain access to a heat source, we conducted two analyses using linear mixed effects models. The first model included the heat trial *z*-score as the dependent variable. The second model included the average number of surrounding ducks (defined as the number of ducklings making direct contact with each duckling) as the dependent variable, to examine how central each duckling’s position was in the brood regardless of proximity to the heat source. For both of these models, incubation temperature was the independent variable, brood and nest ID (nest that eggs were collected from) were included as random effects, and body mass (gram), sex, lay date, age (hours old), and room temperature were included as covariates.

We built linear mixed effects models to answer two questions for each feeding trial: (1) what factors affect feeding behavior (*z*-score)? and (2) after taking feeding behavior (*z*-score) into account, is incubation temperature related to food consumption? For the first question, feeding behavior (*z*-score) was the dependent variable (separate model for each of the three feeding trials). For these models, incubation temperature was the independent variable and brood and nest ID were included as random effects. Duckling age (hours) at the time of the trial, body mass (gram), lay date, sex, and the ambient temperature during the trial were all included as covariates. For the second question, the change in body mass during the 1-h trial (i.e., body mass after–body mass before trial) was the dependent variable, feeding behavior (*z*-score) and incubation temperature were the independent variables (separate model for each of the three feeding trials), and brood and nest ID were included as random effects. We also originally included the interaction between feeding behavior and incubation temperature in all models, but subsequently dropped it because it was not significant in any model (all *P *≥* *0.12). After finding that incubation temperature did indeed explain variance in food consumption that was not explained by feeding behavior (see “Results” section), we also added duckling culmen length as a covariate because we predicted that differences in culmen length might result in differences in food consumption independently of behavior. We excluded two extreme and influential (>20 times the mean Cook’s distance) outliers from our models and figures, one from the novel environment feeding trial and one from the familiar environment feeding trial.

To investigate effects of incubation temperature on duckling body mass, tarsus length, and culmen length throughout the experiment, we constructed three linear mixed effects models. Because we used the same ducklings to measure these three aspects of morphology, we used a Bonferroni correction (α = 0.05/3 = 0.0167) and thus set significance at *P *<* *0.0167 for all models investigating morphology. For two of these models, either body mass (gram) or culmen length (millimeter) was the dependent variable and data were included for Days 0, 2, 4, 6, 8, and 10. For the third model, tarsus length (mm) was the dependent variable and data were included for Days 0, 3, 6, 8, and 10. Only ducklings that survived until Day 10 were used in these morphological analyses (35°C: *n *=* *54; 36°C: *n *=* *54 ducklings). For all models, incubation temperature, age (days; categorical), and their interaction were included as independent variables. Duckling ID was included as a random effect to account for repeated measures and nest ID was included to correct for potential non-independence of eggs collected from the same nest. We investigated pairwise comparisons using estimated marginal means, using the *emmeans* (Lenth 2018) package.

## Results

### Hatch success and incubation period

Hatch success (%) and incubation periods (days) were within the range of values observed in other studies of wood duck eggs artificially incubated at similar temperatures (Hepp et al. 2006; DuRant et al. 2010, 2012a, 2012b, 2013a, 2016; Hope et al. 2018b). Hatch success was 62% for eggs incubated at 35.0°C and 74% for those incubated at 36.0°C. Average (±SD) incubation periods were 38.5 ± 0.9 d for eggs incubated at 35.0°C and 35.7 ± 1.0 d for those incubated at 36.0°C.

### Gaining access to a heat source

**Fig. 1:**
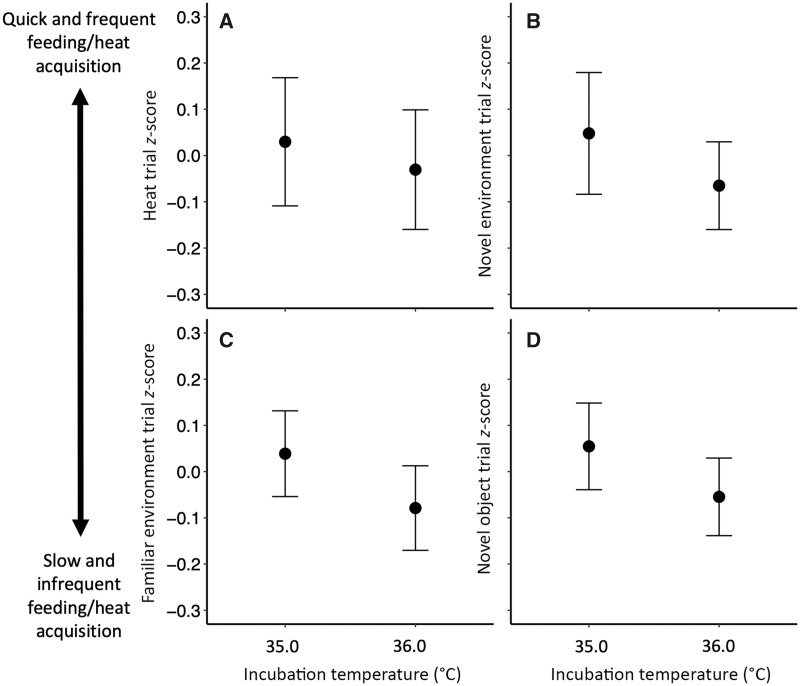
Incubation temperature did not affect duckling behavior (mean *z*-score ± SE) in relation to gaining access to (**A**) heat, (**B**) food in a novel environment, (**C**) food in a familiar environment, or (**D**) food with a novel object placed next to it. Trials were conducted on mixed-incubation temperature duckling broods (2–3 ducklings from each incubation temperature treatment per brood).

Contrary to our predictions, incubation temperature did not influence duckling behaviors related to seeking a concentrated heat source, either in the model investigating the relationship of incubation temperature to *z*-score (*P *=* *0.47; Fig. 1A;*n *=* *15 broods; 45 ducklings from 35.0°C, 44 ducklings from 36.0°C; Supplementary Table S1) or the model investigating its relationship to the average number of surrounding ducklings (*P *=* *0.85; *n *=* *19 broods; 57 ducklings from 35.0°C, 56 ducklings from 36.0°C; Supplementary Table S2). There were also no significant covariates retained in either model (Supplementary Tables S1 and S2).

### Food acquisition in different contexts

Contrary to our predictions, duckling feeding behavior (*z*-score) was not affected by incubation temperature in any of the three trials (effect of incubation temperature: all *P *≥* *0.23; Fig. 1B–D; all trials *n *=* *19 broods; novel environment: *n *=* *55 ducklings from 35.0°C, 55 ducklings from 36.0°C; familiar environment: *n *=* *53 ducklings from 35.0°C, 55 ducklings from 36.0°C; novel object: *n *=* *54 ducklings from 35.0°C, 54 ducklings from 36.0°C; Supplementary Tables S3–S5). However, in both the familiar environment and novel object feeding trials, duckling body mass was negatively related to feeding behavior (*z*-score) (familiar environment: *X*^2^ = 7.60, *P *=* *0.006, Supplementary Table S4; novel object: *X*^2^ = 7.99, *P *=* *0.005, Supplementary Table S5) and thus, individuals with a greater body mass spent slightly less time feeding than those with a lower body mass. However, the correlation coefficients for both of these relationships were quite low (familiar environment: *r* = −0.19; novel object: *r* = −0.11), and thus the relationships between body mass and behavior were not strong.

**Fig. 2:**
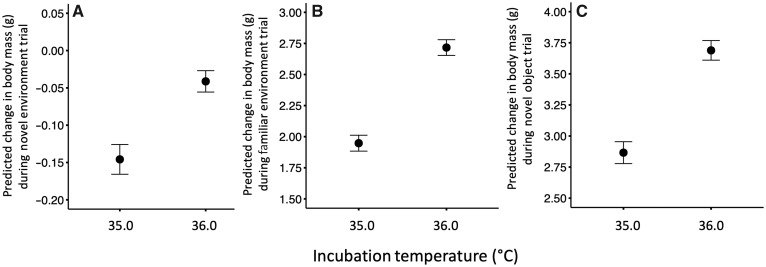
Ducklings incubated at 36.0°C consumed more food than those incubated at 35.0°C during the (**A**) novel environment, (**B**) familiar environment, and (**C**) novel object feeding trials. Change in body mass during the 1-h trials (mass after–mass before trial) is indicative of food consumption. Points are mean ± SE. Predicted body masses were generated using simple linear models with feeding behavior (*z*-score) as a covariate, and thus take this significant covariate into account. Trials were conducted on mixed-incubation temperature duckling broods (2–3 ducklings from each incubation temperature treatment per brood).

**Fig. 3:**
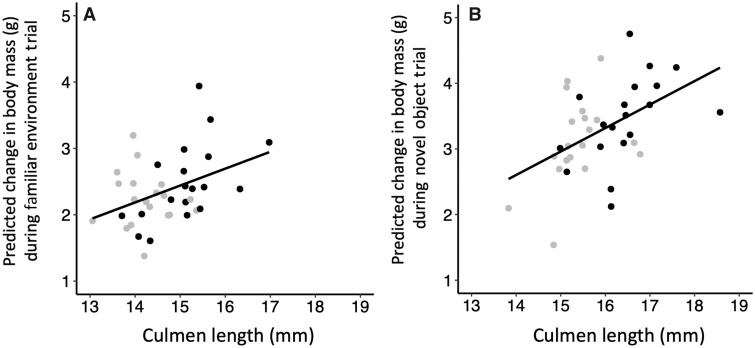
Culmen length was positively related to change in body mass (g) during the familiar environment (**A**) and novel object (**B**) feeding trials. Change in body mass during the 1-h trials (mass after–mass before trial) is indicative of food consumption. Predicted body masses were generated using simple linear models with feeding behavior (*z*-score) as a covariate, and thus take this significant covariate into account. Trials were conducted on mixed-incubation temperature duckling broods (2–3 ducklings from each incubation temperature treatment per brood). For simplicity, data from ducklings incubated at the same temperature are pooled within broods for this figure (*n *=* *19 broods), although data analyses were conducted using brood as a random effect. Point color indicates the temperature at which ducklings were incubated (gray = 35.0°C; black = 36.0°C).

Incubation temperature had a significant (or marginally significant, after the Bonferroni correction) effect on apparent food consumption in all three trials (novel environment: *P *=* *0.013, Fig 2A, Supplementary Table S6; familiar environment: *P *<* *0.0001, Fig 2B, Supplementary Table S7; novel object: *P *<* *0.0001, Fig. 2C, Supplementary Table S8) where, in all cases, given the same feeding behavior, high temperature-incubated ducklings consumed more food during a trial than low temperature-incubated ducklings. Culmen length was positively related to apparent food consumption in the familiar environment (*P *<* *0.0001, Fig. 3A, Supplementary Table S7) and novel object trials (*P *<* *0.0001, Fig. 3B, Supplementary Table S8), but not the novel environment trial (*P *=* *0.28; Supplementary Table S6). Taken together, these results suggest that the difference in apparent food consumption during the 1-h feeding trials among ducklings incubated at different temperatures could be driven, at least in part, by differences in structural size.

### Body mass, structural size, and growth

**Fig. 4:**
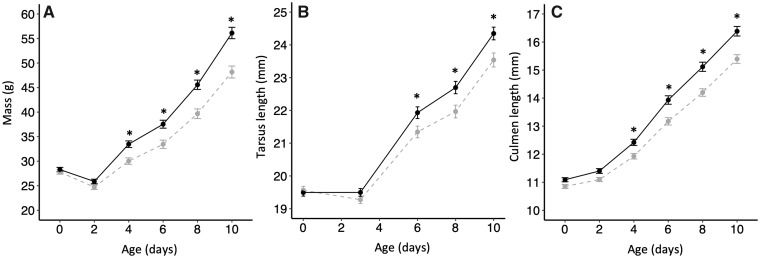
(**a**) Body mass (mean ± SE), (**B**) tarsus length (mean ± SE), and (**C**) culmen length (mean ± SE) of ducklings incubated at either 35°C (gray) or 36°C (black) from hatch (Day 0) until Day 10. Note that tarsus was measured on Day 3 instead of Days 2 and 4. *There was a significant difference between incubation temperatures.

There was an interactive effect of incubation temperature and age (days) on body mass (incubation temperature: *X*^2^ = 0.22, *P *=* *0.64; age: *X*^2^ = 1780, *P *<* *0.0001; interaction: *X*^2^ = 94.5, *P *<* *0.0001; Supplementary Table S9; Fig. 4A), tarsus length (incubation temperature: *X*^2^ < 0.001, *P *=* *0.99; age: *X*^2^ = 1245, *P *<* *0.0001; interaction: *X*^2^ = 27.2, *P *<* *0.0001; Supplementary Table S10; Fig. 4B), and culmen length (incubation temperature: *X*^2^ = 1.96, *P *=* *0.16; age: *X*^2^ = 3340, *P *<* *0.0001; interaction: *X*^2^ = 51.9, *P *<* *0.0001; Supplementary Table S11; Fig. 4C). Pairwise comparisons among ages revealed that shortly after hatching, all ducklings lost body mass, grew larger culmens, and had no change in tarsus length (pairwise comparisons: Days 0–2: mass: *P* < 0.001; culmen: *P* = 0.0009; Days 1–3: tarsus: *P* = 0.62; Fig. 4). After that (Days 4–10), ducklings grew larger in all aspects of morphology as they aged (all pairwise comparisons: *P* < 0.0001; Fig. 4). As for the interaction with incubation temperature, pairwise comparisons revealed that ducklings incubated at different temperatures were of similar size during the first few days, and then those incubated at the higher temperature grew faster until the end of the experiment. Specifically, there were no differences between ducklings incubated at different temperatures in body mass on Day 0 or 2, tarsus length on Day 0 or 3, or culmen length on Day 0 or 2 (all *P *≥* *0.08). However, ducklings incubated at the higher temperature had greater body masses on Days 4–10 (all *P *≤* *0.003), longer tarsus lengths on Days 6–10 (all *P *≤* *0.013), and longer culmen lengths on Days 4–10 (all *P *≤* *0.005), than those incubated at the lower temperature (Fig. 4).

## Discussion

In this study, we investigated whether incubation temperature affects acquisition of food and heat resources within broods of precocial avian offspring. Because incubation temperature influences multiple fitness-related offspring traits in birds (reviewed in DuRant et al. 2013b) and average incubation temperatures vary among eggs within nests (Hope et al. 2018a), we predicted that this could create differences among brood mates in the ability to acquire resources. Contrary to our predictions, we found no difference in the behaviors related to food or heat acquisition among ducklings incubated at different temperatures when tested in mixed-incubation temperature broods. However, ducklings incubated at the higher temperature consumed more food during the 1-h feeding trials and had greater body mass and structural size than those incubated at the lower temperature. Thus, our results suggest that individuals incubated at low temperatures may be disadvantaged compared to brood mates incubated at higher temperatures, but this deficit is likely due to differences in structural size rather than behaviors, at least after ducklings are ∼5 days old.

Consistent with previous studies (reviewed in DuRant et al. 2013b), we found evidence that ducklings incubated at low temperatures displayed a physiological deficit related to their growth rates compared to those incubated at higher temperatures. Ducklings incubated at different temperatures had similar body masses and structural sizes until Day 2–3, after which ducklings incubated at the higher temperature had greater body masses and longer tarsus and culmen lengths than those incubated at the lower temperature until the end of the study (Day 10). Furthermore, ducklings incubated at the higher temperature consistently consumed more food during the 1-h feeding trials than those incubated at the lower temperature. Because the frequency of feeding behavior did not differ among treatments, the differences in apparent food consumption between ducklings incubated at different temperatures appeared to be driven by differences in duckling structural size, rather than by differences in behavior. Specifically, because food consumption was related to culmen length, it is likely that the larger bills of ducklings incubated at high temperatures helped them to consume more food per bite than their counterparts incubated at a cooler temperature, similar to the relationships found between gape size and food consumption across other taxa (e.g., Wheelwright 1985; Singha et al. 2015; Luiz et al. 2019). This could create a positive feedback loop, wherein larger ducklings are more efficient at consuming food, which leads to faster growth rates and the ability to consume even more food. This positive feedback could amplify differences in phenotype among brood mates incubated at different temperatures and, in part, underlie the incubation temperature-induced differences in body size and growth in this study, and in previous studies (DuRant et al. 2010; Nord and Nilsson 2011; Wada et al. 2015; Ospina et al. 2018).

Our observations suggest that ducklings incubated at higher temperatures may have an advantage compared to brood mates that experienced slightly lower incubation temperatures, which could have important consequences in a natural setting. For example, although all ducklings exhibited a similar capacity to secure time near a heat source, ducklings incubated at the higher temperature would likely have a greater chance of surviving cold conditions than those incubated at the lower temperature because of the inherent thermoregulatory advantages of a larger body size (Rhymer 1988) and because they expend less energy and maintain higher body temperatures during a thermoregulatory challenge (DuRant et al. 2012b, 2013a). Similarly, although incubation temperature did not affect the frequency of feeding behavior in our trials, it is likely that the ability of ducklings incubated at the higher temperature to consume more food per feeding bout would allow them to gain access to more food compared to those incubated at the lower temperature when food is limited. A greater feeding efficiency could also allow ducklings to feed more quickly, limiting their time spent in the open and vulnerable to predators. Furthermore, the larger body mass of a high temperature-incubated duckling could increase the chances of recovering from a period of mass loss (Arroyo 2002) or decrease the chances of predation by gape-limited predators (e.g., fish). Indeed, a recent meta-analysis found that offspring body mass generally has a positive relationship with offspring survival across mammal and bird species (Ronget et al. 2018), and a study on wood ducks also found that survival probability in the wild increased with duckling body mass (Sedinger et al. 2018). Because 50–75% of wood duck mortality in the wild occurs within the first 1–2 weeks of life (McGilvrey 1969; Sedinger et al. 2018), the effect of incubation temperature on body mass, growth, structural size, and food consumption in 4–10 day-old ducklings could give high temperature-incubated ducklings an early advantage in the most critical days of life. This may explain, in part, why studies have found evidence that avian offspring incubated at higher temperatures have higher long-term survival compared to those incubated at lower temperatures (zebra finches: Berntsen and Bech 2016; wood ducks: Hepp and Kennamer 2012). However, it is important to note that a larger body size could also be disadvantageous in some cases (Blanckenhorn 2000). For example, larger bodies have higher metabolic demands, which would be disadvantageous if food is difficult to find. The complexity of the relationship between offspring body mass and survival could be the reason why one study found that nestlings incubated at high temperatures with large body masses experienced lower survival compared to smaller nestlings (blue tits; Nord and Nilsson 2016). Further, although our experimental trials provided limited access to heat (single small source) and food (small scattered sources, or single small source), food and heat availability in the wild are likely more limited and unpredictable. Thus, future studies are needed to fully link the results from our study to consequences in the wild.

Incubation is a parental effect that can influence the behavioral, physiological, and morphological phenotype of the individual (DuRant et al. 2013b), and the phenotypic composition of the brood through variation in average incubation temperature within nests (Hope et al. 2018a). Although there has been accumulating evidence over the past decade that incubation temperature affects diverse avian offspring traits, this is the first study to investigate whether these trait differences could be amplified or reduced due to the phenotypic composition of the brood. Our study provides evidence that higher incubation temperatures lead to larger body sizes, which in turn lead to increased efficiency of food consumption, rather than differences in frequency of feeding behavior. This provides insight into how an important avian parental effect could generate positive feedback that amplifies early phenotypic differences among offspring within broods. In altricial species, there is ample research that suggests that parental effects can create differences in offspring growth and size through hatching asynchrony, hormone deposition, or differential food allocation, leading to differential nestling survival within broods (Schwabl 1996; Ostreiher 1997; Krebs et al. 1999; Ploger and Medeiros 2004; Morandini and Ferrer 2015). Our study reveals a previously unrecognized way by which differential survival among offspring could occur post-fledging, potentially within both altricial and precocial broods.

## Supplementary Material

obaa003_Supplementary_Data
