# Supplementary material for: Incubation Temperature Affects Duckling Body Size and Food Consumption Despite No Effect on Associated Feeding Behaviors
Source: Integr Org Biol. 2020 Feb 5;2(1):obaa003. doi: 10.1093/iob/obaa003 (PMC7671149; doi:10.1093/iob/obaa003)
Supplement: obaa003_Supplementary_Data [file obaa003_supplementary_data.docx]

**SUPPLEMENTARY INFORMATION**

**Supplementary Tables**

| **Dependent variable: Heat Trial z-score**  *N = 15 broods; 45 ducklings from 35.0°C, 44 ducklings from 36.0°C* | | |
| --- | --- | --- |
| **Full Model** | | |
| Term | *X^2^* | *P* |
| Incubation temperature | 0.53 | 0.47 |
| Body mass | 0.97 | 0.33 |
| Sex | 0.12 | 0.73 |
| Lay date | 3.86 | 0.0495 |
| Age (hours) | 1.04 | 0.31 |
| Room temperature | 0.43 | 0.51 |
| **Final Model** |  |  |
| *No significant terms* |  |  |

**Table S1.** Full and reduced linear mixed effects model investigating whether incubation temperature and other covariates influence duckling behavior when attempting to gain access to a heat source. Ducklings were in groups of 6 (3 incubated at 35°C and 3 incubated at 36°C). Brood and the nest that eggs were collected from were included as random effects. The dependent variable was duckling behavior (z-score), where a higher z-score indicated that the duckling was quick to go to the heat and spent a large quantity of time under the heat. The model was reduced using stepwise backward elimination of insignificant terms (p > 0.0125; Bonferroni correction for reusing same individuals in 4 behavioral trials).

| **Dependent variable: Heat Trial Average number of ducks surrounding**  *N = 19 broods; 57 ducklings from 35.0°C, 56 ducklings from 36.0°C* | | |
| --- | --- | --- |
| **Full Model** | | |
| Term | *X^2^* | *P* |
| Incubation temperature | 0.04 | 0.85 |
| Body mass | 1.72 | 0.19 |
| Sex | 0.04 | 0.83 |
| Lay date | 2.66 | 0.10 |
| Age (hours) | 0.60 | 0.44 |
| Room temperature | 0.15 | 0.70 |
| **Final Model** |  |  |
| *No significant terms* |  |  |

**Table S2.** Full and reduced linear mixed effects model investigating whether incubation temperature and other covariates influence duckling behavior when attempting to gain access to a heat source. The dependent variable was the average number of ducklings that were surrounding each duckling (i.e., measure of optimal, central position within the brood). Ducklings were in groups of 6 (3 incubated at 35°C and 3 incubated at 36°C). Brood and the nest that eggs were collected from were included as random effects. The model was reduced using stepwise backward elimination of insignificant terms (p > 0.0125; Bonferroni correction for reusing same individuals in 4 behavioral trials).

| **Dependent variable: Novel Environment Feeding Trial z-score**  *N = 19 broods; 55 ducklings from 35.0°C, 55 ducklings from 36.0°C* | | |
| --- | --- | --- |
| **Full Model** | | |
| Term | *X^2^* | *P* |
| Incubation temperature | 1.42 | 0.23 |
| Body mass | 2.22 | 0.14 |
| Sex | 0.11 | 0.74 |
| Lay date | 0.14 | 0.71 |
| Age (hours) | 0.14 | 0.71 |
| Ambient temperature | 0.35 | 0.56 |
| **Final Model** |  |  |
| *No significant terms* |  |  |

**Table S3.** Full and reduced linear mixed effects model investigating whether incubation temperature and other covariates influence duckling behavior in relation to gaining access to food in a novel environment. The dependent variable was feeding behavior (z-score), where a high z-score indicates that the duckling was quick to begin feeding and fed frequently. Body mass was measured before the trial. Ducklings were in groups of 6 (3 incubated at 35°C and 3 incubated at 36°C). Brood and the nest that eggs were collected from were included as random effects. The model was reduced using stepwise backward elimination of insignificant terms (p > 0.0125; Bonferroni correction for reusing same individuals in 4 behavioral trials).

| **Dependent variable: Familiar Environment Feeding Trial z-score**  *N = 19 broods; 53 ducklings from 35.0°C, 55 ducklings from 36.0°C* | | |
| --- | --- | --- |
| **Full Model** | | |
| Term | *X^2^* | *P* |
| Incubation temperature | 0.86 | 0.35 |
| Body mass | 4.93 | 0.026 |
| Sex | 0.94 | 0.33 |
| Lay date | 2.26 | 0.13 |
| Age (hours) | 0.0003 | 0.99 |
| Ambient temperature | 2.96 | 0.09 |
| **Final Model** |  |  |
| Body mass | **7.60** | **0.006** |

**Table S4.** Full and reduced linear mixed effects model investigating whether incubation temperature and other covariates influence duckling behavior in relation to gaining access to food in a familiar environment. The dependent variable was feeding behavior (z-score), where a high z- score indicates that the duckling was quick to begin feeding and fed frequently. Body mass was measured before the trial. Ducklings were in groups of 6 (3 incubated at 35°C and 3 incubated at 36°C). Brood and the nest that eggs were collected from were included as random effects. The model was reduced using stepwise backward elimination of insignificant terms (p > 0.0125; Bonferroni correction for reusing same individuals in 4 behavioral trials).

| **Dependent variable: Novel Object Feeding Trial z-score**  *N = 19 broods; 54 ducklings from 35.0°C, 54 ducklings from 36.0°C* | | |
| --- | --- | --- |
| **Full Model** | | |
| Term | *X^2^* | *P* |
| Incubation temperature | 1.30 | 0.25 |
| Body mass | 3.79 | 0.052 |
| Sex | 0.10 | 0.76 |
| Lay date | 0.61 | 0.43 |
| Age (hours) | 0.03 | 0.86 |
| Ambient temperature | 0.71 | 0.40 |
| **Final Model** |  |  |
| Body mass | **7.99** | **0.005** |

**Table S5.** Full and reduced linear mixed effects model investigating whether incubation temperature and other covariates influence duckling behavior in relation to gaining access to food that had a novel object placed next to it. The dependent variable was feeding behavior (z-score), where a high z-score indicates that the duckling was quick to begin feeding and fed frequently. Body mass was measured before the trial. Ducklings were in groups of 6 (3 incubated at 35°C and 3 incubated at 36°C). Brood and the nest that eggs were collected from were included as random effects. The model was reduced using stepwise backward elimination of insignificant terms (p > 0.0125; Bonferroni correction for reusing same individuals in 4 behavioral trials).

| **Dependent variable: Novel Environment Feeding Trial change in body mass**  *N = 19 broods; 55 ducklings from 35.0°C, 55 ducklings from 36.0°C* | | |
| --- | --- | --- |
| **First Model** | | |
| Term | *X^2^* | *P* |
| Feeding behavior (z-score) | **11.7** | **0.0006** |
| Incubation temperature | 6.13 | 0.013 |
| **Second Model (including culmen)** |  |  |
| Feeding behavior (z-score) | **11.1** | **0.0009** |
| Incubation temperature | 4.00 | 0.046 |
| Culmen length | 1.15 | 0.28 |

**Table S6.** Linear mixed effects model investigating whether feeding behavior (z-score) is an accurate indication of food consumption (change in body mass) during a trial where ducklings seek access to food in a novel environment. A high z-score indicates that the duckling was quick to begin feeding and fed frequently. Incubation temperature was included as a covariate to investigate whether there might be an effect of incubation temperature on food consumption that was not explained by behavioral differences. Because of the results of the first model, culmen length (mm) was included as a covariate in the second model in an attempt to explain differences in food consumption. Ducklings were in groups of 6 (3 incubated at 35°C and 3 incubated at 36°C). Brood and the nest that eggs were collected from were included as random effects. Significance was defined as p < 0.0125; Bonferroni correction for reusing same individuals in 4 behavioral trials.

| **Dependent variable: Familiar Environment Feeding Trial change in body mass**  *N = 19 broods; 53 ducklings from 35.0°C, 55 ducklings from 36.0°C* | | |
| --- | --- | --- |
| **First Model** | | |
| Term | *X^2^* | *P* |
| Feeding behavior (z-score) | **6.30** | **0.0121** |
| Incubation temperature | **20.6** | **<0.0001** |
| **Second Model (including culmen)** |  |  |
| Feeding behavior (z-score) | **9.73** | **0.002** |
| Incubation temperature | **9.43** | **0.002** |
| Culmen length | **16.4** | **<0.0001** |

**Table S7.** Linear mixed effects model investigating whether feeding behavior (z-score) is an accurate indication of food consumption (change in body mass) during a trial where ducklings seek access to food in a familiar environment. A high z-score indicates that the duckling was quick to begin feeding and fed frequently. Incubation temperature was included as a covariate to investigate whether there might be an effect of incubation temperature on food consumption that was not explained by behavioral differences. Because of the results of the first model, culmen length (mm) was included as a covariate in the second model in an attempt to explain differences in food consumption. Ducklings were in groups of 6 (3 incubated at 35°C and 3 incubated at 36°C). Brood and the nest that eggs were collected from were included as random effects. Significance was defined as p < 0.0125; Bonferroni correction for reusing same individuals in 4 behavioral trials.

| **Dependent variable: Novel Object Feeding Trial change in body mass**  *N = 19 broods; 54 ducklings from 35.0°C, 54 ducklings from 36.0°C* | | |
| --- | --- | --- |
| **First Model** | | |
| Term | *X^2^* | *P* |
| Feeding behavior (z-score) | **12.8** | **0.0003** |
| Incubation temperature | **17.7** | **<0.0001** |
| **Second Model (including culmen)** |  |  |
| Feeding behavior (z-score) | **20.2** | **<0.0001** |
| Incubation temperature | 6.18 | 0.0129 |
| Culmen length | **20.5** | **<0.0001** |

**Table S8.** Linear mixed effects model investigating whether feeding behavior (z-score) is an accurate indication of food consumption (change in body mass) during a trial where ducklings seek access to food that had a novel object placed next to it. A high z-score indicates that the duckling was quick to begin feeding and fed frequently. Incubation temperature was included as a covariate to investigate whether there might be an effect of incubation temperature on food consumption that was not explained by behavioral differences. Because of the results of the first model, culmen length (mm) was included as a covariate in the second model in an attempt to explain differences in food consumption. Ducklings were in groups of 6 (3 incubated at 35°C and 3 incubated at 36°C). Brood and the nest that eggs were collected from were included as random effects. Significance was defined as p < 0.0125; Bonferroni correction for reusing same individuals in 4 behavioral trials.

| **Dependent variable: Body mass (g)**  *N = 108 ducklings; 648 measurements* | | |
| --- | --- | --- |
| **Full Model** | | |
| Term | *X^2^* | *P* |
| Incubation temperature | 0.22 | 0.64 |
| Age (day) | **1780** | **<0.0001** |
| Incubation temperature x Age | **94.5** | **<0.0001** |

**Table S9.** Linear mixed effects model investigating how duckling body mass changed over time (day 0, 2, 4, 6, 8, and 10) and differed depending on incubation temperature. Duckling ID was included as a random effect because each duckling was measured multiple times. The nest that the eggs were collected from was also included as a random effect. Significance was defined as p < 0.0167; Bonferroni correction for reusing same individuals to investigate 3 aspects of morphology.

| **Dependent variable: Tarsus length (mm)**  *N = 108 ducklings; 540 measurements* | | |
| --- | --- | --- |
| **Full Model** | | |
| Term | *X^2^* | *P* |
| Incubation temperature | <0.001 | 0.99 |
| Age (day) | **1245** | **<0.0001** |
| Incubation temperature x Age | **27.2** | **<0.0001** |

**Table S10.** Linear mixed effects model investigating how duckling tarsus length changed over time (day 0, 3, 6, 8, and 10) and differed depending on incubation temperature. Duckling ID was included as a random effect because each duckling was measured multiple times. The nest that the eggs were collected from was also included as a random effect. Significance was defined as p < 0.0167; Bonferroni correction for reusing same individuals to investigate 3 aspects of morphology.

| **Dependent variable: Culmen length (mm)**  *N = 108 ducklings; 648 measurements* | | |
| --- | --- | --- |
| **Full Model** | | |
| Term | *X^2^* | *P* |
| Incubation temperature | 1.96 | 0.16 |
| Age (day) | **3340** | **<0.0001** |
| Incubation temperature x Age | **51.9** | **<0.0001** |

**Table S11.** Linear mixed effects model investigating how duckling culmen length changed over time (day 0, 2, 4, 6, 8, and 10) and differed depending on incubation temperature. Duckling ID was included as a random effect because each duckling was measured multiple times. The nest that the eggs were collected from was also included as a random effect. Significance was defined as p < 0.0167; Bonferroni correction for reusing same individuals to investigate 3 aspects of morphology.

**
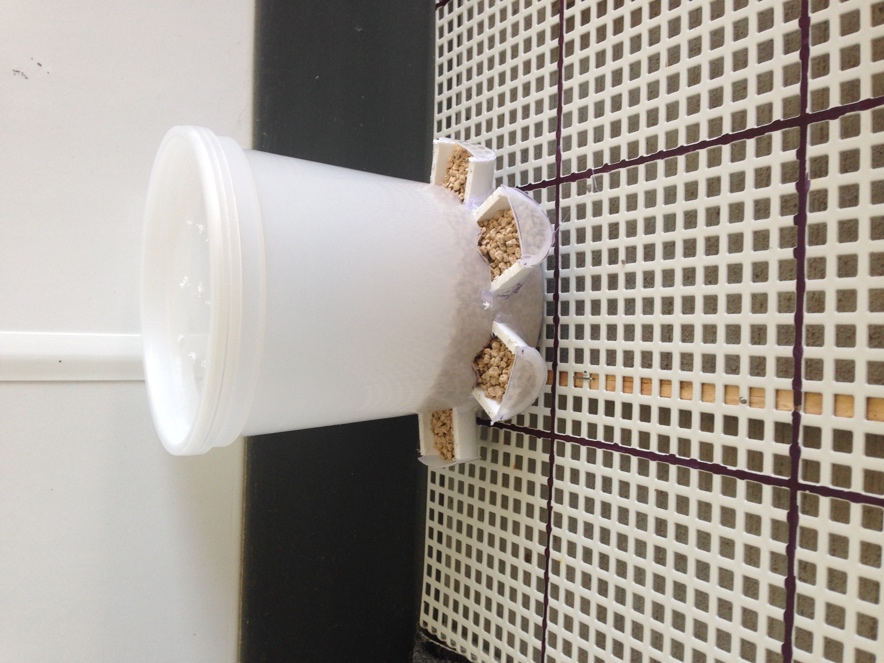

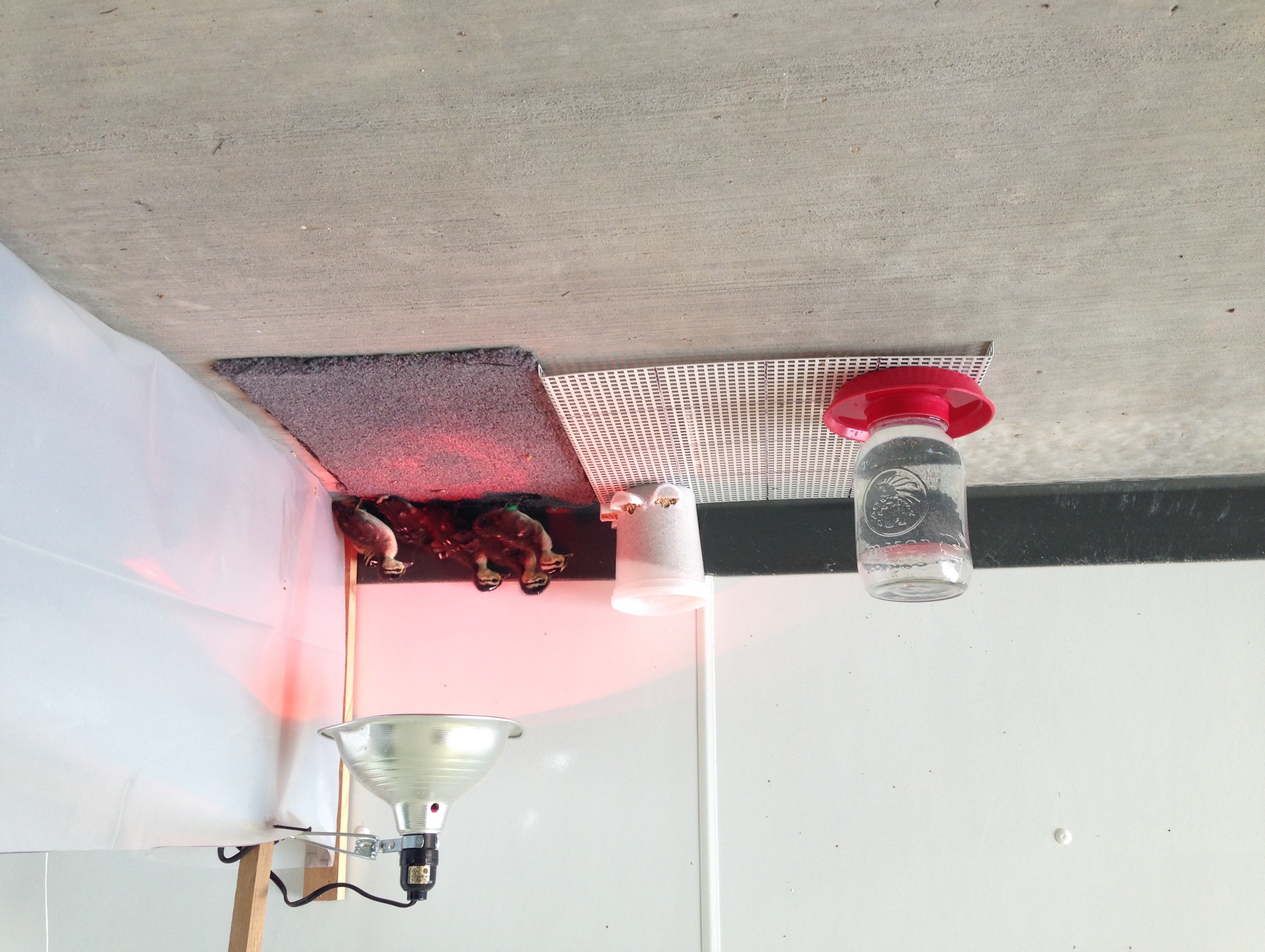

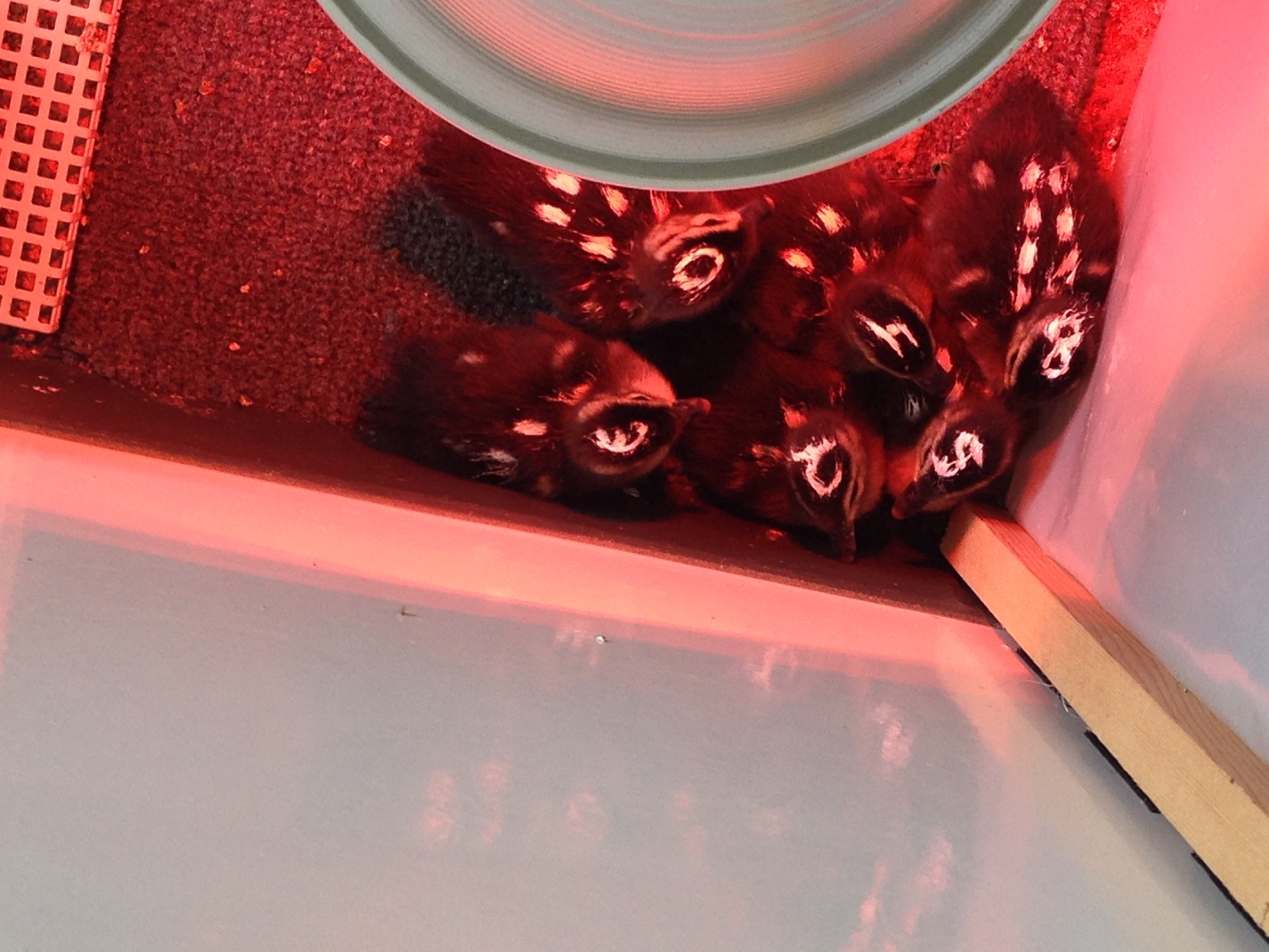
Supplementary Figures**

**B**

**A**

**C**

**Figure S1.** **Daily ducking housing set-up.** (A) Ducklings were numbered for individual recognition in behavioral trials. (B) The food dish used daily had multiple openings and resembled the food dish that was used in feeing trials (Fig. S4). (C) There was a grate under the food dish so ducklings could not eat food spilled on the floor during feeding trials.


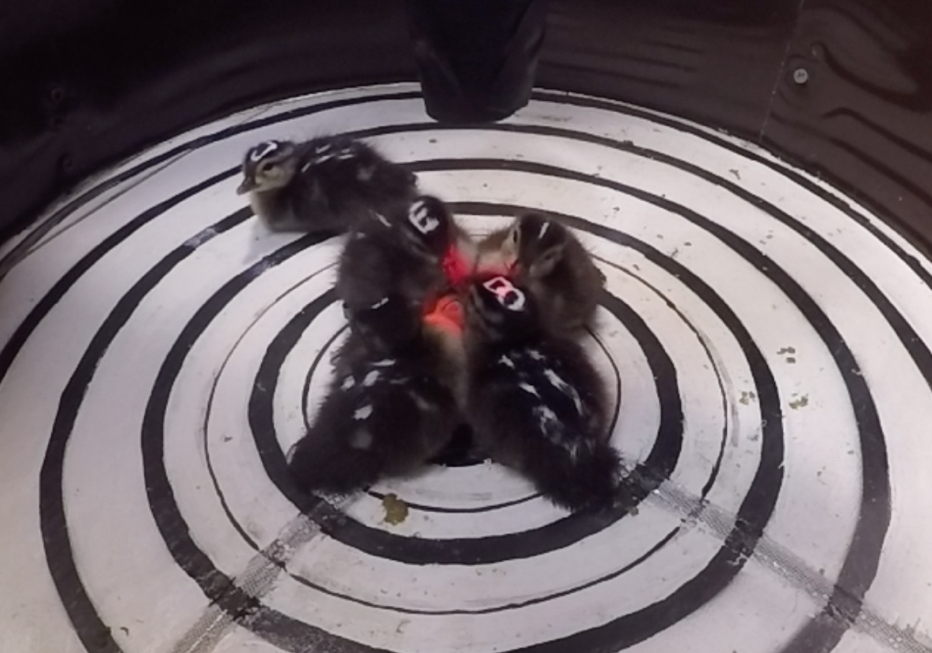


**A**


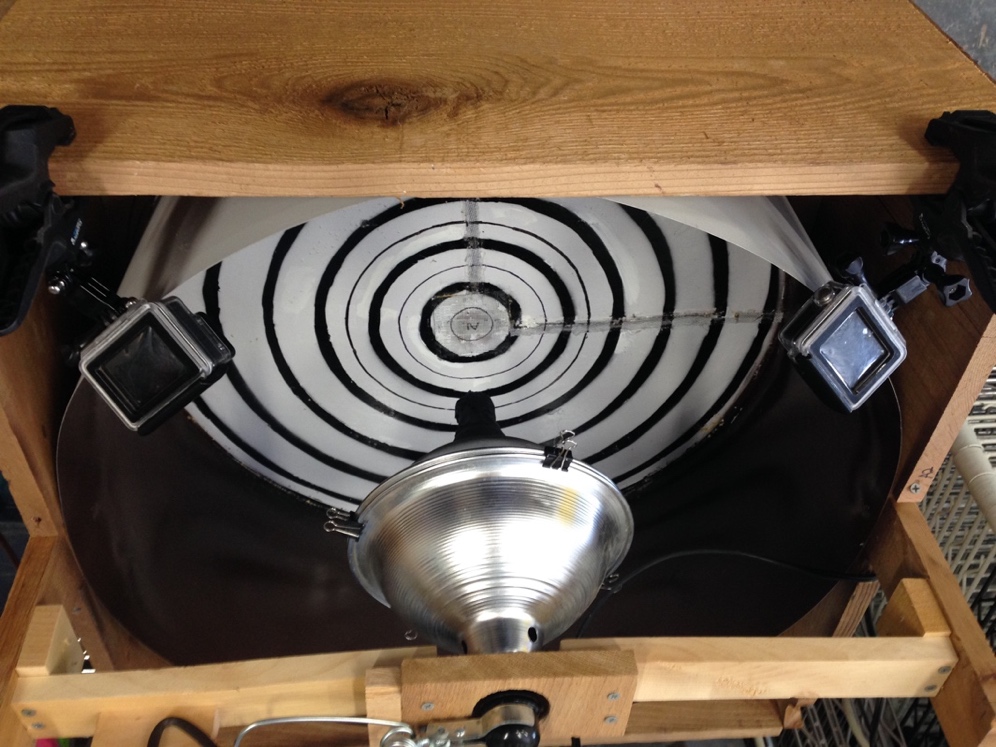


**B**

**Figure S2. The Heat Trial arena.** (A) Ducklings attempted to gain access to a centralized heat source (infrared heat lamp). Lines in the trial arena were used to calculate the position of each duckling during the trial, where a lower position number indicated that the duckling was closer to the heat source. The position was determined based on the circle in which the majority of the duckling’s body was located. Diameter of central position was 4 cm. Length between positions 2, 3, 4, 5, and 6 was 2 cm. For the three most exterior circles, there was a space of 4 cm between each circle, and thus these were given positions of 8, 10, and 12. (B) A zoomed-out picture of the trial arena; GoPro^©^ cameras were used to video record the trials.

**Figure S3. Schematic of Heat Trial.** Aerial view of the Heat Trial. Numbers represent approximate temperatures during the trial. Red lines = distance of 4 cm; yellow line = 8 cm; blue line = 14 cm.


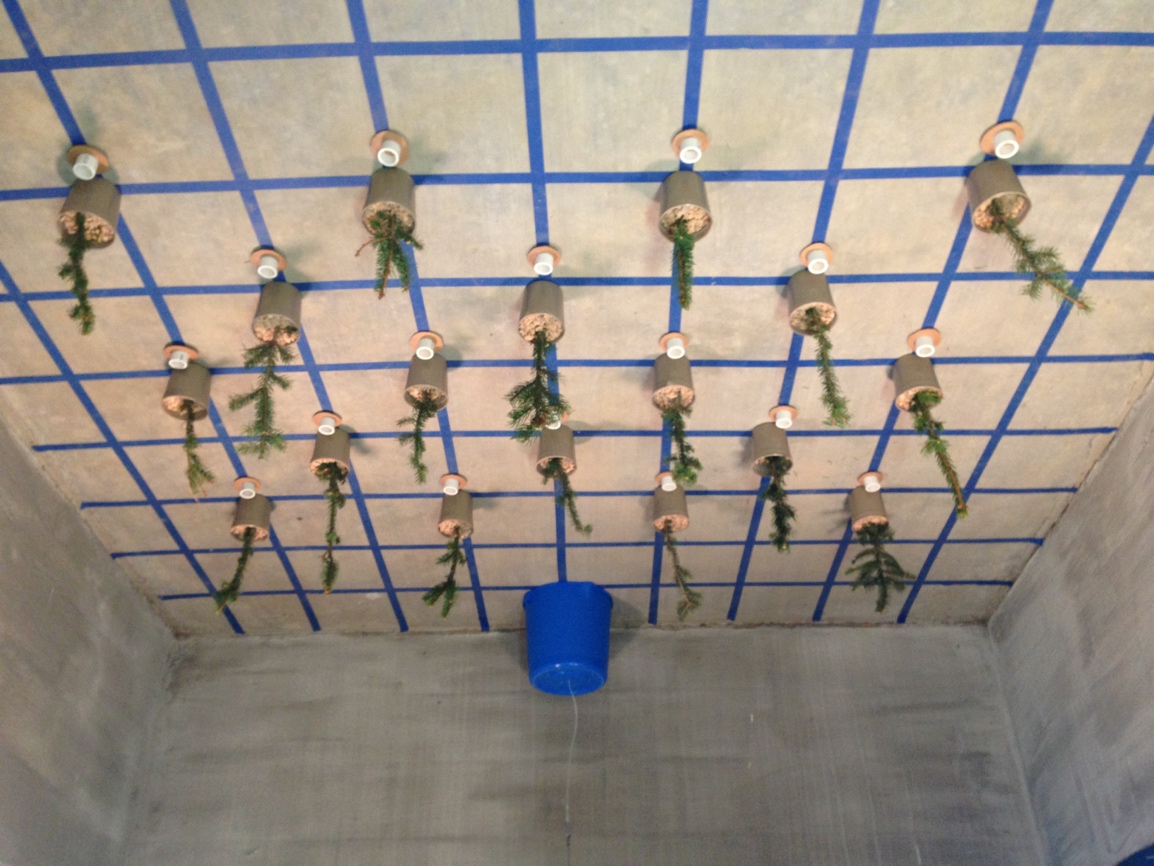


**Figure S4. The Novel Environment Feeding Trial arena.** Ducklings acclimated in the blue bucket, then explored the arena (novel environment) to find food. Food was placed in white dishes, each behind a small potted plant.


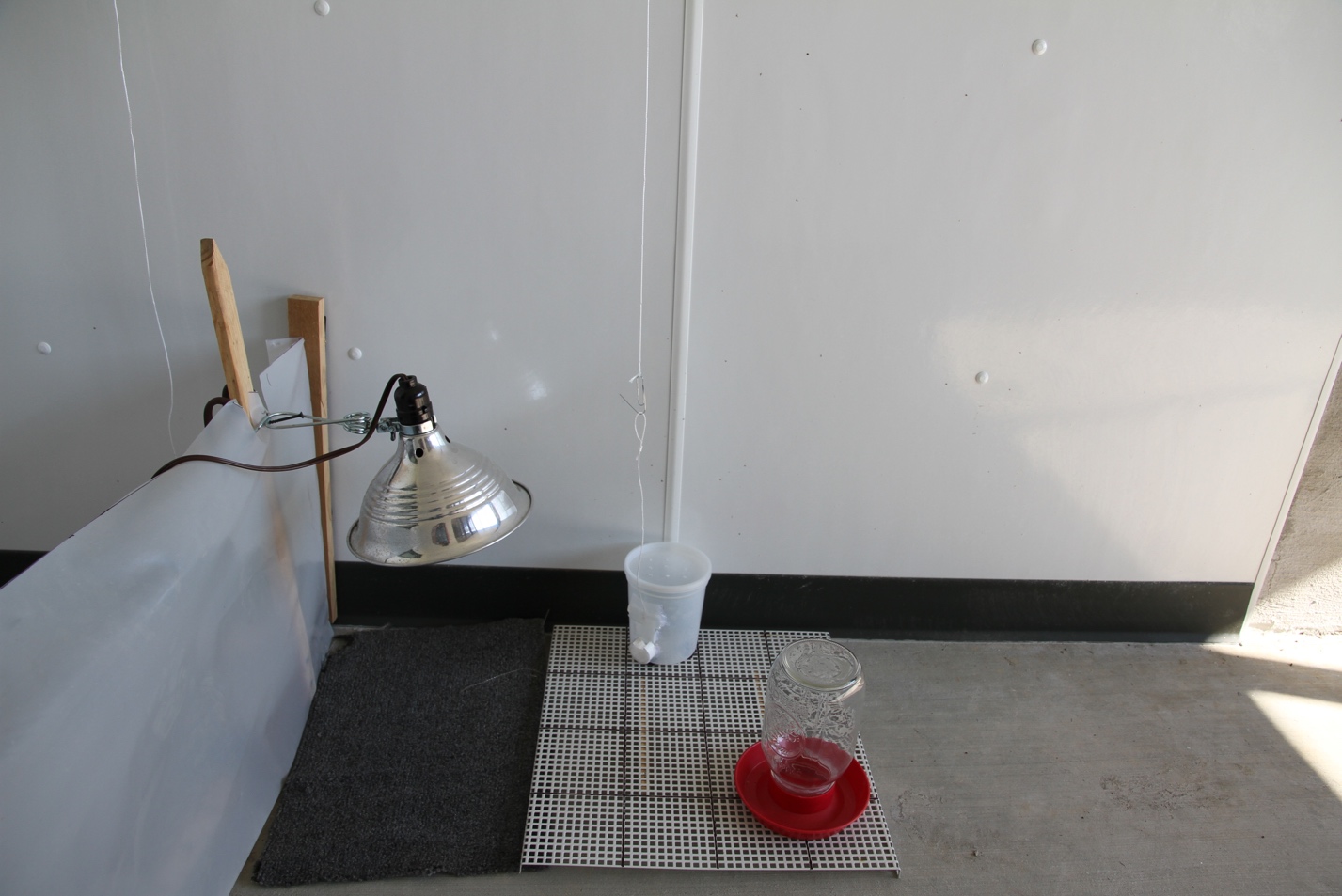

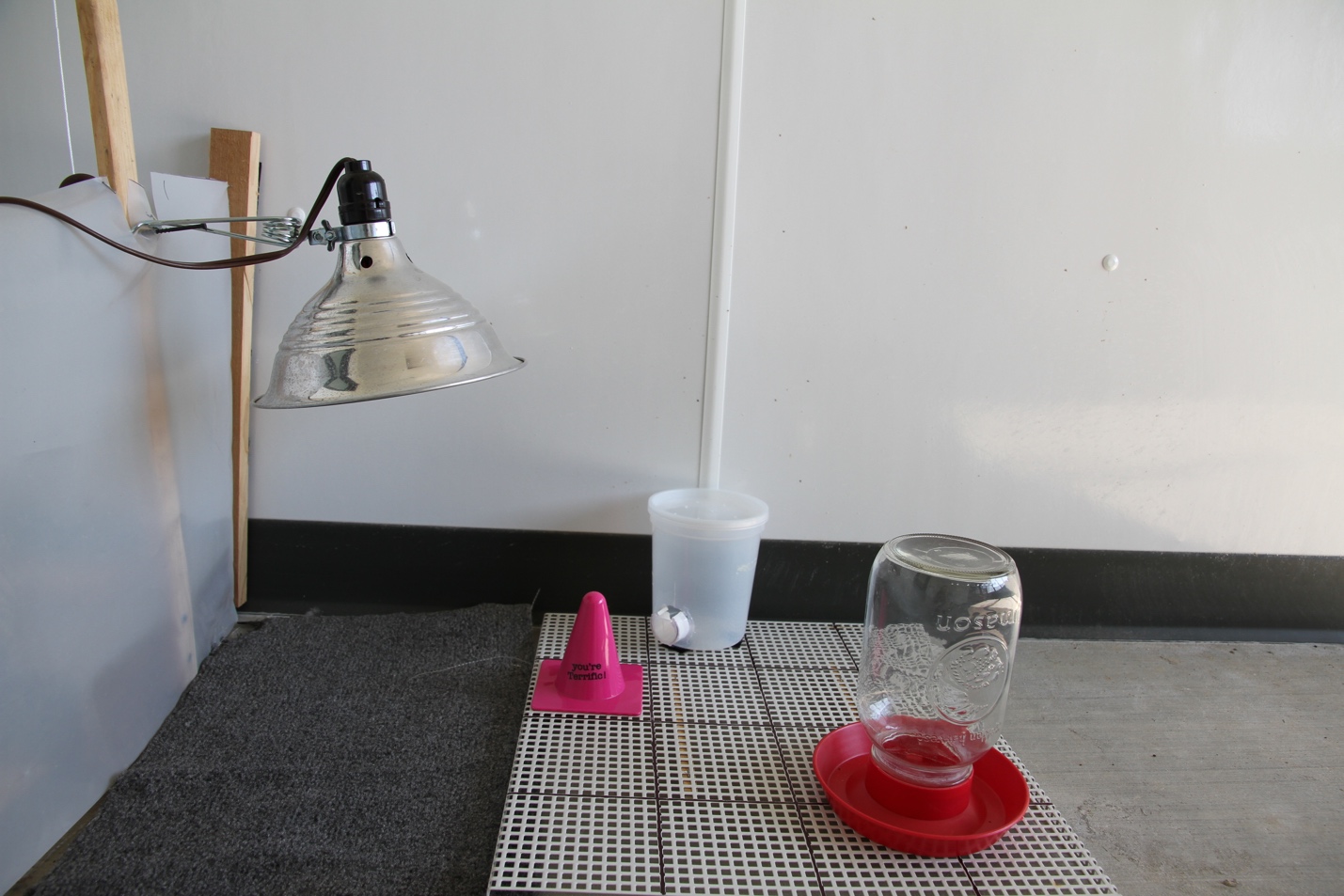


**B**

**A**

**Figure S5. Familiar Environment and Novel Object Feeding Trial set-ups.** (A) The Familiar Environment Feeding Trial was conducted in the home aviary (i.e., familiar environment). The normal food dish (Fig. S1B) was replaced by a similar food dish, but with only one opening for food. A string was used to remotely reveal food after letting ducklings acclimate. (B) The Novel Object Feeding Trial was the same as the Familiar Environment Feeding Trial, but there was a novel object (pink cone) placed next to the food.
